# P06-11 Continuity and changes in commuting mode and influence on physical activity, BMI and waist circumference among Finnish adults

**DOI:** 10.1093/eurpub/ckac095.096

**Published:** 2022-08-29

**Authors:** Salin Kasper, Tuomas Kukko, Kaisa Kaseva, Tuija Tammelin, Xiaolin Yang, Olli T Raitakari, Harto Hakonen

**Affiliations:** 1 Faculty of Sport & Health Sciences, University of Jyväskylä, Jyväskylä, Finland; 2 JAMK University of Applied Sciences, Jyväskylä, Finland; 3 University of Turku, Turku, Finland

**Keywords:** Active commuting, physical activity, objective measurement, BMI

## Abstract

**Background:**

Regular physical activity (PA) has been found to be important for cardiovascular health and longevity. However, notable proportion of adult population does not meet the national PA recommendations. Active transport is one domain of physical activity, that could be a time-efficient way to increase PA and reach the national recommendations. Additionally, it could have a positive effect to body composition.

**Methods:**

Based on longitudinal cohort study, active commuting modes and objectively measured PA were used to determine the influence of commuting mode to steps, aerobic steps, BMI and waist circumference. Linear regression models were fitted to test the associations between the change groups of commuting mode and the longitudinal changes of the response variables.

**Results:**

When compared to passive commuters, participants with public transport (p = 0.09) and walking (p > 0.001-0.021) showed higher amounts of steps and aerobic during summertime and wintertime. Cyclers showed higher amounts of steps and aerobic steps only in wintertime (p = 0.001-0.002). Passive commuters had higher BMI than walkers (p = 0.05) and cyclers (p = 0.023) in summertime. Also, cyclers had lower waist circumference than passive commuters (p = 0.016-0.02). Among those who remained persistently active, number of steps did not change. When compared to persistently active, among those who changed from active to passive commuting, steps (-900 - -885) and aerobic steps (-500) declined (p = 0.010-0.036) while among those who changed from passive to active commuting steps (+900-1000) and aerobic steps (+650-750) increased (p = 0.023-0.011).

**Conclusions:**

Commuting actively to work and changing passive mode to active mode has a positive effect to number of daily steps and aerobic steps. Since the active commuting is part of the daily routine, promoting active commuting could be one of the key factors tackling the obesity and insufficient PA among adults. Continuity of active commuting seemed to be effective way to maintain body weight in balance over the years.

